# Predictive value of the preoperative neutrophil-to-lymphocyte ratio for the development of hepatocellular carcinoma in HBV-associated cirrhotic patients after splenectomy

**DOI:** 10.1371/journal.pone.0195336

**Published:** 2018-04-05

**Authors:** Zhaoqing Du, Jian Dong, Jianbin Bi, Ruhai Bai, Jia Zhang, Zheng Wu, Yi Lv, Xufeng Zhang, Rongqian Wu

**Affiliations:** 1 Shaanxi Provincial Center for Regenerative Medicine and Surgical Engineering, Xi’an, Shaanxi Province, China; 2 Institute of Advanced Surgical Technology and Engineering, Xi’an Jiaotong University, Xi’an, Shaanxi Province, China; 3 Department of Hepatobiliary Surgery,First Affiliated Hospital, Xi’an Jiaotong University, Xi’an, Shaanxi Province, China; 4 Department of Epidemiology and Biostatistics, Xi’an Jiaotong University School of Public Health, Xi’an, Shaanxi Province, China; University of Navarra School of Medicine and Center for Applied Medical Research (CIMA), SPAIN

## Abstract

The neutrophil to lymphocyte ratio (NLR), a simple marker of inflammation, has recently been showed to predict tumor recurrence in hepatocellular carcinoma (HCC) patients after hepatic resection or liver transplantation. However, whether it can be used to predict HCC development in cirrhotic patients remained unknown. The aim of this study was to evaluate the predictive value of the preoperative NLR for the development of HCC in cirrhotic patients who underwent splenectomy. A total of 230 HBV-associated cirrhotic patients who underwent splenectomy in our hospital from January 2000 to December 2012 were included in this study. Detailed clinical data included patients’ general characteristics, laboratory tests and imaging studies, surgical procedures and complications. Information on patients’ follow-up data was also obtained. We found that 38 (16.52%) patients developed HCC after splenectomy during the follow-up period. An elevated preoperative NLR was associated with increased risk of developing HCC in cirrhotic patients after splenectomy. The optimal cutoff value of NLR for HCC development was 2.27. In patients who developed HCC during the follow-up period, NLR scores showed no predictive value in overall survival after splenectomy. However, NLR scores appeared to have a much better predictive value in overall survival in patients who did not develop HCC. In conclusion, cirrhotic patients who underwent splenectomy remain at a relatively high risk of developing HCC, and an elevated preoperative NLR is associated with HCC development in cirrhotic patients who underwent splenectomy for hypersplenism.

## Introduction

Hepatocellular carcinoma (HCC) is one of the most common malignant tumors in the world. Although methods for detecting and treating HCC have been greatly improved, its mortality is still high[1,2]. Cirrhosis is a major risk factor for HCC. Approximately 70%-90% of HCC patients have underlying cirrhosis. Hypersplenism is a common complication of liver cirrhosis. A recent study has shown that hypersplenism is positively correlated with the increased risk of HCC in patients with post-hepatitis cirrhosis[3]. Splenectomy improved anti-tumor immune mechanisms and reduced HCC risk. However, many cirrhotic patients who underwent splenectomy still developed HCC. Inflammatory responses play a vital role in tumorigenesis[4]. The development of HCC is closely associated with inflammation status and immune responses[5]. The neutrophil to lymphocyte ratio (NLR) is a simple marker of inflammation. It provides a good indication of the patient’s overall inflammatory status. An elevated NLR has been associated with increased mortality in several malignancies[6–12]. Recently, NLR has been used to predict tumor recurrence in HCC patients after hepatic resection[13] or liver transplantation[14]. However, there are no published data assessing the role of NLR in predicting HCC development in cirrhotic patients after splenectomy. The main purpose of this study, therefore, was to determine whether an elevated NLR is associated with HCC development in cirrhotic patients who underwent splenectomy for hypersplenism, and if so, to determine the optimal predictive NLR cutoff value. The primary endpoint was the appearance of HCC after splenectomy and the secondary endpoint was the overall survival. Since the majority of cirrhotic patients in China have history of HBV infection, we focused on HBV-associated cirrhotic patients in the current study.

## Materials and methods

### Study population

This retrospective cohort study was conducted to investigate the relationship between NLR and the development of HCC in cirrhotic patients after splenectomy. The inclusion criteria were: 1) underwent splenectomy at the first affiliated hospital of Xi'an Jiaotong University from January 2000 to December 2012, 2) history of chronic hepatitis B virus (HBV) infection, 3) the presence of liver cirrhosis with hypersplenism but without tumor at the time of splenectomy, and 4) available medical records including follow-up data. Ultimately 230 patients were included in this study. The study has been approved by the First Affiliated Hospital of Xi'an Jiaotong University Ethics Committee. The patient’s informed written consent was waived due to the retrospective nature of this study. All data collected were used only for statistics analysis in this study.

### Data collection

Detailed clinical data included patients’ age, sex, length of hospital stay, admission diagnosis, past history, Child-Pugh class/score and other general clinical data, as well as biochemical and imaging studies, surgical procedures and complications. Laboratory tests included blood routine test, liver function, renal function and blood coagulation indicators, which were documented at admission. The sizes of the spleen volume were assessed by imaging instrumentation. The preoperative data were obtained prior to surgery, and the blood samples were taken on the first day after admission. The first set of measurements was taken if there were more than one set for the same individual. NLR was calculated as neutrophils divided by the absolute number of lymphocytes[15].

### Patient follow-up and HCC diagnosis

All cases received follow-up routinely until June 2015. Overall incidence of HCC and overall survival were calculated from the time of surgery in our unit. The time of occurrence of HCC was defined as the time from the surgery to the last observation of no tumor. The follow-up data was achieved every three months in the first year, every four months in the second year, every six months in the third year, and every one year thereafter. The content included abdominal symptoms and signs, recurrence of gastrointestinal bleeding, hepatitis B virus controlling, AFP values, Doppler ultrasound examination and abdominal computed tomography results. Based on the corresponding symptoms and related inspection materials, we determined whether there was a tumor. During the period of follow-up, patient-related data, including death and gastrointestinal bleeding, tumor occurrence and imaging examination, had been recorded in detail. To minimize bias, two clinicians completed follow-up and review respectively. The diagnosis of HCC was based on past history, clinical symptoms and signs, laboratory tests and imaging results.

### Statistical analysis

For continuous variables, the data was recorded with mean ± standard deviation or median (minimum—maximum), which was based on whether they meet the distributed Kolmogorov-Smirnov test, (*p*<0.05) or not. Categorical variables were shown as frequency. Therefore, the significance of difference between two groups was detected by calculating the Student’s *t*-test or Wilcoxon test for continuous datum and the Chi-squared test for categorical variables. The receiver operating characteristic (ROC) curve with overall incidence of HCC was used to decide the ideal cutoff value, allowing the indication for overall HCC occurrence with the optimal sensitivity and specificity. Survival curves were plotted using the Kaplan-Meier method, and the differences between the groups were analyzed by the Log-Rank test. If there were significant statistical differences in the univariate analysis, the multivariate Cox proportional hazards model was further performed. The whole statistical process was completed by the PASW Statistics 22.0 software (IBM Corporation, Armonk, NY, USA). While the drawing of survival curve were accomplished by the Graphpad prism 6.0 software (GraphPad Software, Inc. La Jolla, USA). And the subgroup analysis was performed after excluding the confounding factors. In this study, *p* values<0.05 was considered statistically significant.

## Results

### Patient demographics and characteristics

A total of 230 HBV-associated cirrhotic patients who underwent splenectomy in our hospital from January 2000 to December 2012 were included in this study. As shown in Table 1, the median age was 44 years (range: 20–66 years) and 174 were male (75.65%). Patients' lifestyle showed that 10.87% had history of alcohol use, 23.48% had history of smoking. Hypertension, hyperlipidemia, and diabetes mellitus were seen in 21.30%, 20.87%, and 6.96% of the patients, respectively. On admission, 199 (86.52%) patients had gastro-esophageal varices, 108 (46.96%) patients had ascites, and 1 (0.43%) patient had hepatic encephalopathy. As for the Child-Pugh class, 57 (24.78%) patients were in class A, 155 (67.39%) in class B, and 18 (7.83%) in class C. During the surgery, the median blood loss was 470ml (range: 50–2000), and the median blood transfusion was 600ml (range: 400–1400). The median volume of resected spleens was 1426mm^3^ (range: 196–6800). After splenectomy, 2 (0.87%) patients had intra-abdominal active bleeding, 34 (14.78%) patients developed portal vein or splenic vein thrombosis. Ascites formation was seen in 35 (15.22%) patients. Six (2.61%) patients developed intra-abdominal infection, 1 (0.43%) patient had surgical site infection, and 3 (1.30%) patients had pneumonia after surgery. During hospitalization, 1 (0.43%) patient had a cerebrovascular accident. 204 patients (88.70%) underwent open surgery and no patients died during hospitalization. The average follow-up period was 65 months and was as long as 185 months. The overall survival rate was 84.3%.

**Table 1:** Clinical characteristic of the 230 patients.

Patient Demographics and Clinical Characteristics	Median(range)/n(percentage)
N	230
Age(years)	44(20–66)
Gender (male: female)	174:56
Coexisting conditions	
Drinking	25(10.87%)
Smoking	54(23.48%)
Hypertension	49(21.30%)
Diabetes	16(6.96%)
Gastro-esophageal varices	199(86.52%)
Child-Pugh Score	7(5–12)
Child A	57(24.78%)
Child B	154(66.96%)
Child C	18(7.83%)
Unavailable	1(0.43%)
Estimated blood loss (ml)	470(50–2000)
Intraoperative transfusion (ml)	600(400–1400)
Spleen volume (mm^3^)	1426(196–6800)
Hemorrhage after surgery	2(0.87%)
Portal vein thrombosis	34(14.78%)
Ascites after surgery	35(15.22%)
Infection	10(4.35%)
Cerebrovascular accident	1(0.43%)
Surgical approach	
Open splenectomy	204(88.70%)
Laparoscopy	26(11.30%)
In-hospital Death	0(0%)
Overall Survival	194(84.35%)

### Preoperative NLR score and HCC development

**Fig 1:**
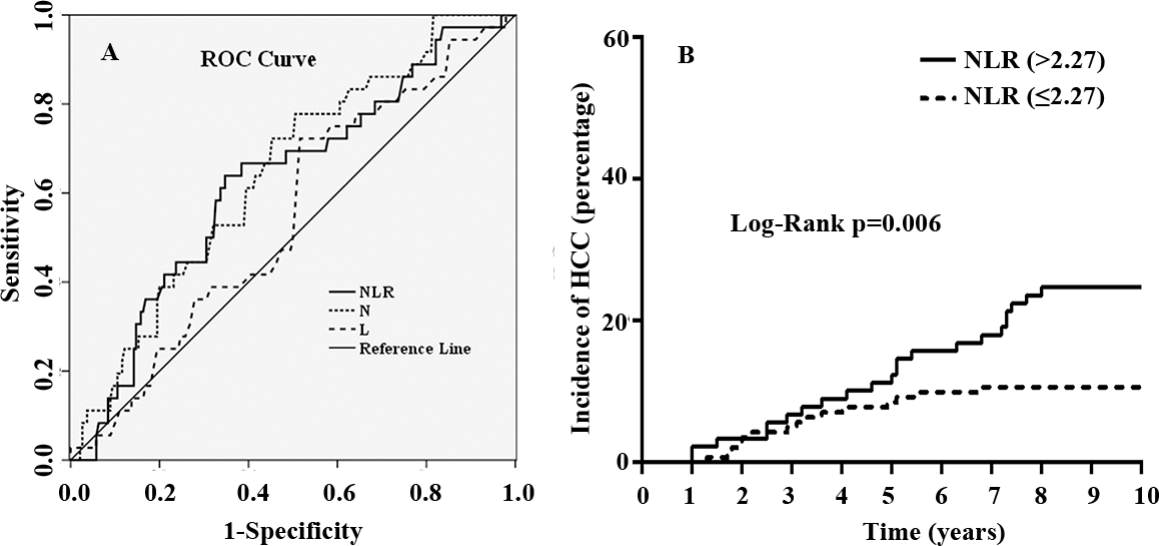
Preoperative neutrophil-to-lymphocyte ratio (NLR) and hepatocellular carcinoma (HCC) development in cirrhotic patients after splenectomy. **A.** a receiver operating characteristic (ROC) curve analysis of the predictive value of the preoperative NLR scores on hepatocarcinogenesis after splenectomy. The area under the ROC curve (AUC) was 0.625 (95% confidence interval, 0.527–0.723, P = 0.018). The calculated cut-off for NLR scores to predict HCC development was 2.27, with sensitivity of 63.9%, specificity of 65.3%, and Youden index of 0.292; **B.** a Kaplan-Meier plot for the association of the preoperative NLR score with the cumulative incidence of HCC after splenectomy. The difference between the two groups was compared by the Log-Rank test.

During the follow-up period, 38 (16.52%) out of 230 patients with HBV-related cirrhosis developed HCC after splenectomy. The cumulative HCC appearance rates were 6.09%, 10.87% and 17.39% at 3, 5 and 10 years after splenectomy, respectively. To determine the value of the preoperative NLR score in predicting hepatocarcinogenesis after splenectomy, an ROC curve analysis was performed. As shown in Fig 1A, the preoperative NLR score had a positive correlation with the HCC appearance rate after splenectomy. The area under the ROC curve (AUC) was 0.625 (95% confidence interval, 0.527–0.723, P = 0.018). The calculated cut-off for NLR scores to predict HCC development was 2.27, with sensitivity of 63.9%, specificity of 65.3%, and Youden index of 0.292. According to the cut-off value of the preoperative NLR score, 141 patients in this cohort had a low NLR score (NLR≤2.27, 61.3%) and 89 patients had a high NLR score (NLR>2.27, 38.7%). Table 2 shows the demographic and preoperative data associated with high or low NLR scores. Compared with patients with a low NLR score, patients with a high NLR score had a higher neutrophil count (P<0.001), but a lower lymphocyte count (P<0.001). However, there were no statistically significant differences in other baseline demographic and clinical parameters. The Kaplan-Meier estimates of HCC appearance rates after splenectomy were calculated with regard to preoperative NLR scores. As shown in Fig 1B, the 3, 5 and 10 years HCC appearance rates were 6.7%, 12.4%, and 24.7% in the high NLR group and 5.0%, 8.5%, and 10.6% in the low NLR group, respectively. The difference was statistically significant (P = 0.006).

**Table 2:** Clinical characteristics of patients based on NLR.

Variables	NLR (0–2.27)	NLR (>2.27)	*p* value
n	141	89	
Gender (male)	109(77.30%)	65(73.03%)	0.462
Age	44(21–66)	42(20–64)	0.077
Coexisting conditions			
Drinking	18(12.77%)	7(7.87%)	0.245
Smoking	35(24.82%)	19(21.35%)	0.545
Hypertension	26(18.44%)	23(25.84%)	0.182
Diabetes	12(8.51%)	4(4.49%)	0.296
Neutrophil count (10^9^/L)	1.07(0.08–3.38)	2.30(0.55–8.71)	**<0.001**
Lymphocyte count (10^9^/L)	0.73(0.21–1.78)	0.58(0.07–2.00)	**<0.001**
Platelet count (<30×10^9^/L)	51(36.17%)	25(28.09%)	0.204
Albumin (<35g/L)	67(47.52%)	44(49.44%)	0.776
ALT (>40U/L)	50(35.46%)	33(37.08%)	0.804
AST (>40U/L)	67(47.52%)	48(53.93%)	0.343
Creatinine (umol/L)	67.44(15.00–132.90)	68.41(32.00–187.60)	0.690
PT (s)	16.3(11.0–25.6)	16.5(12.2–27.2)	0.669
APTT (s)	44(23–88)	45(29–95)	0.512
INR (>1.2)	95(67.38%)	64(71.91%)	0.468
AFP	12.17(0.68–125.80)	9.37(0.61–140.90)	0.359
Child-Pugh Score			0.616
Child A	32(22.70%)	25(28.09%)	
Child B	97(68.79%)	57(64.04%)	
Child C	11(7.80%)	7(7.87%)	
Unavailable	1(0.71%)	0(0%)	
Spleen volume (mm^3^)	1470(196–6800)	1355(360–3150)	0.322
Surgical approach (open splenectomy/laparoscopy)	79/10	125/16	0.979
HCC occurrence (yes/no)	15/126	23/66	**0.002**

Abbreviations: ALT, alanine aminotransferase; AST, aspartate transaminase; PT, prothrombin time; APTT, activated partial thromboplastin time; INR, international normalized ratio; AFP, alpha fetoprotein; HCC, hepatocellular carcinoma.

### Risk factors for hepatocarcinogenesis

To identify risk factors independently associated with hepatocarcinogenesis after splenectomy, univariable and multivariable analyses were performed. Univariate variables with P<0.1 were entered into the multivariate model. As shown in Table 3, only the preoperative NLR score was independently associated with hepatocarcinogenesis after splenectomy in the multivariate analysis. The odds ratio of patients with a NLR score of >2.27 was 3.304 compared with those with a lower NLR score. Other factors including age, sex, platelet count<30×10^9^/L, AFP levels, Child-Pugh class, intraoperative blood loss or blood transfusion, postoperative bleeding, portal vein thrombosis, the spleen size, surgical approach, and postoperative complications were not independently associated with the hepatocarcinogenesis rate.

**Table 3:** Univariable and multivariable analysis of risk factors for the development of HCC.

Parameters	Univariate analysis		Multivariate analysis	
	*p* value	OR (95% CI)	*p* value	OR (95% CI)
Age>50 years	0.534	1.294(0.575–2.910)		
Gender (male/female)	0.917	0.957(0.423–2.167)		
Smoking (yes/no)	0.652	1.201(0.542–2.665)		
Drinking (yes/no)	0.522	0.662(0.188–2.335)		
Ascites (yes/no)	0.513	0.791(0.391–1.597)		
Hypertension (yes/no)	0.211	1.651(0.752–3.623)		
NLR (high/low)	**0.002**	3.324(1.582–6.986)	**0.004**	3.304(1.477–7.394)
Platelet count (<30×10^9^/L)	0.315	0.697(0.345–1.409)		
ALT (>40U/L)	0.235	1.533(0.757–3.105)		
AST (>40U/L)	**0.016**	2.483(1.184–5.208)	0.065	2.147(0.954–4.834)
INR (>1.2)	0.051	2.965(0.994–8.841)	0.088	2.656(0.866–8.150)
AFP	0.622	1.004(0.987–1.022)		
Child-Pugh Score (Child C *vs*. Child A)	0.305	2.041(0.522–7.983)		
Child-Pugh Score (Child B *vs*. Child A)	0.359	1.519(0.621–3.710)		
Estimated blood loss ≥470ml	0.985	1.015(0.208–4.965)		
Intraoperative transfusion (yes/no)	0.917	1.040(0.492–2.202)		
Spleen volume ≥1426mm^3^	0.832	0.992(0.921–1.069)		
Portal vein thrombosis (yes/no)	0.743	0.849(0.319–2.257)		
Surgical approach (open splenectomy *vs*. laparoscopy)	0.169	0.353(0.080–1.555)		
Postoperative complications (yes/no)	0.515	1.277(0.611–2.668)		

Abbreviations: NLR, neutrophil-to-lymphocyte ratio; ALT, alanine aminotransferase; AST, aspartate transaminase; INR, international normalized ratio; AFP, alpha fetoprotein.

### Preoperative NLR score and overall survival

**Fig 2:**
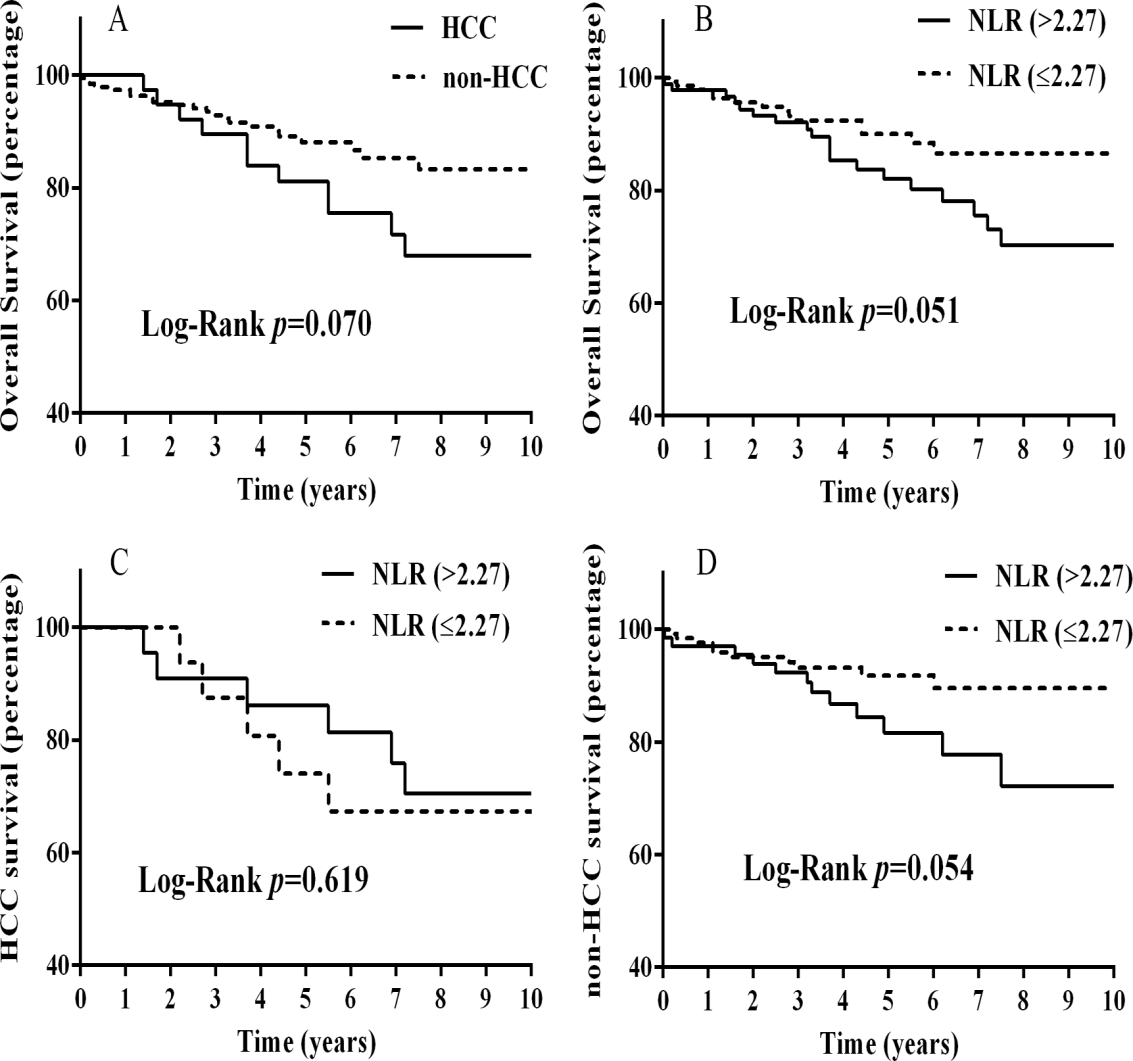
Preoperative neutrophil-to-lymphocyte ratio (NLR) and overall survival after splenectomy. **A.** difference in survival rates in cirrhotic patients who developed HCC after splenectomy (HCC) and those who did not develop HCC (non-HCC); **B.** difference in survival rates in cirrhotic patients who had a preoperative NLR >2.27 and those who had a NLR ≤2.27; **C.** difference in survival rates in HCC patients who had a preoperative NLR >2.27 and those who had a NLR ≤2.27; **D.** difference in survival rates in non-HCC patients who had a preoperative NLR >2.27 and those who had a NLR ≤2.27. The survival rate was estimated by the Kaplan-Meier method and compared by the Log-Rank test.

During the follow-up period, a total of 36 (15.65%) patients died. The 3-, 5- and 10-year survival rates were 92.6%, 88.7%, and 85.7%, respectively. The patients who developed HCC had a lower overall survival rate than those who did not develop HCC. However, the difference was not statistically significant (P = 0.07 in the log-rank test, Fig 2A). To determine the value of the preoperative NLR score in predicting survival after splenectomy, the Kaplan-Meier estimates of survival after splenectomy were calculated with regard to their preoperative NLR scores in total patients, HCC patients and non-HCC patients, respectively. In the whole study population, patients with a high NLR score appeared to have a lower overall survival rate than those with a low NLR score after splenectomy (P = 0.051, Fig 2B). However, in patients who developed HCC during the follow-up period, NLR scores showed no predictive value in overall survival after splenectomy (Fig 2C). On the other hand, NLR scores appeared to have a much better predictive value in overall survival after splenectomy in patients who did not develop HCC during the follow-up period (P = 0.054, Fig 2D).

## Discussion

Detecting HCC at the early stage requires accurately identifying the major high-risk population of HCC. Liver cirrhosis is the most important risk factor for HCC development. However, only a small portion of cirrhotic patients eventually develop HCC. The annual incidence of HCC in cirrhotic patients ranges from 0.04% to 6.3%[16–21]. Therefore, methods to distinguish cirrhotic patients with higher and lower risk for HCC development are urgently needed. Here, we found that NLR, an inexpensive and easily accessible marker of inflammation, can be used to assess the risk for the development of HCC in cirrhotic patients and a high preoperative NLR is associated with increased risk of developing HCC in cirrhotic patients who underwent splenectomy.

Splenectomy is a common treatment for cirrhotic patients with hypersplenism and portal hypertension. In a recent study of 2678 cirrhotic patients with hypersplenism, Lv X et al. reported that splenectomy was performed less frequently in patients who developed HCC than those who did not develop HCC, suggesting that splenectomy might reduce HCC risk in cirrhotic patients[3]. However, in that study, 17.3% cirrhotic patients who underwent splenectomy still developed HCC during the follow-up period. In the current study, we also found that the 10-year cumulative HCC appearance rate was 16.1% in cirrhotic patients who underwent splenectomy. Therefore, cirrhotic patients who underwent splenectomy remains at a relatively high risk of developing HCC.

Inflammation plays a crucial role in the development of cancer. NLR is a marker of systemic inflammation. An elevated NLR has been found to be correlated with poor prognosis of HCC patients regardless of the treatment method and the stage of the tumor[14,22–28]. In the current study, we demonstrated for the first time that an elevated preoperative NLR was associated with increased risk of developing HCC in cirrhotic patients after splenectomy. The optimal cutoff value of NLR for HCC development after splenectomy in cirrhotic patients was 2.27. The 10-year cumulative HCC appearance rate in patients with a NLR score > 2.27 was more than double the rate in patients with a NLR score ≤ 2.27 (24.7% vs. 10.6%), suggesting a high risk of HCC in patients with a high NLR score. A recent study has shown that a high NLR score was correlated with accumulation of tumor-associated macrophages in the liver after curative resection of HCC[27]. Tumor-associated macrophages are an important component of the tumor inflammatory microenvironment in HCC and have been shown to promote tumorigenesis[29]. Therefore, the association of the elevated NLR and the increased risk of HCC may be related to the accumulation of tumor-associated macrophages in the liver in such patients. However, the detailed underlying mechanism warrants further investigation.

Here, we also found that the appearance of HCC reduced the lifespan of cirrhotic patients slightly. The 10-year overall survival rate was 71.05% for patients who developed HCC and 86.98% for non-HCC patients. However, the difference was not statistically significant. This is expected as all the patients in this cohort had advanced liver cirrhosis. In order to show a statistically significant difference in survival, more patients were needed. In terms of the predictive value of NLR for the overall survival, it appeared that it performed better in non-HCC patients than HCC patients. This could be due to the fact that when HCC developed in these patients, the whole body’s inflammatory status changed. The NLR before splenectomy could not reflect the inflammatory status after the appearance of HCC, which was the one that is vital for the prognosis of these patients. On the other hand, no such changes in the inflammatory status were undertaken in patients who did not develop HCC. So NLR before splenectomy remained predictive for survival in non-HCC cirrhotic patients. We also noticed that the p values for NLR to predict survival were 0.051 and 0.054 in the whole study cohort and the non-HCC subgroup, respectively. This suggests the optimal cutoff value for predicting HCC development may not be the optimal cutoff value for predicting survival. Nevertheless, more studies aimed at investigating the role of NLR in different groups of cirrhotic patients are needed.

All the patients in this study had chronic HBV infection. High levels of HBV DNA in the circulation are associated with an increased risk of developing HCC[30]. Nucleoside analogues effectively suppress HBV DNA levels. Several studies have shown that long-term nucleoside analogue therapy reduced the risk for developing HBV-related HCC[31–33]. Nucleoside analogue therapy has now become standard treatment modalities for chronic HBV infection. The predictive value of NLR on HBV-related HCC should be evaluated in nucleoside analogue treated HBV patients in the future.

The long follow-up time and the highly focused study population (i.e., HBV-associated cirrhotic patients underwent splenectomy for hypersplenism and portal hypertension) are the obvious strengths of the current study. However, there are also several limitations in this study. First, our data came from a single-center. The sample size was relatively small, which could be the reason for not showing statistically significant differences in the survival analysis. Second, due to the high prevalence of HBV infection in our country, only patients with HBV-related cirrhosis were included in the study. Therefore, whether NLR has the same value in predicting HCC development in patients with HCV or alcohol-related cirrhosis needs to be determined. Third, this is a retrospective cohort study. It is subject to the limitations consistent with this type of studies such as unmeasured or unknown confounding factors and selection biases. A prospective trial may be needed to further validate our findings.

In conclusion, cirrhotic patients who underwent splenectomy remains at a relatively high risk of developing HCC, and an elevated preoperative NLR is associated with HCC development in cirrhotic patients who underwent splenectomy for hypersplenism. NLR may offer additional information in the clinical management of cirrhotic patients with hypersplenism and portal hypertension due to its predictive value in HCC development and long-term survival in those patients.
