# Correction: Predictive value of the preoperative neutrophil-to-lymphocyte ratio for the development of hepatocellular carcinoma in HBV-associated cirrhotic patients after splenectomy

**DOI:** 10.1371/journal.pone.0215183

**Published:** 2019-04-04

**Authors:** Zhaoqing Du, Jian Dong, Jianbin Bi, Ruhai Bai, Jia Zhang, Zheng Wu, Yi Lv, Xufeng Zhang, Rongqian Wu

There are errors in the author affiliations. The correct affiliations are as follows:

Zhaoqing Du^1,2,3^, Jian Dong^1,2,3^, Jianbin Bi^1,2,3^, Ruhai Bai^4^, Jia Zhang^1,2,3^, Zheng Wu^3^, Yi Lv^1,2,3^, Xufeng Zhang^1,2,3^, Rongqian Wu^1,2^

**1** Shaanxi Provincial Center for Regenerative Medicine and Surgical Engineering, First Affiliated Hospital of Xi’an Jiaotong University, Xi’an, Shaanxi Province, China, **2** Institute of Advanced Surgical Technology and Engineering, First Affiliated Hospital of Xi’an Jiaotong University, Xi’an, Shaanxi Province, China, **3** Department of Hepatobiliary Surgery, First Affiliated Hospital of Xi’an Jiaotong University, Xi’an, Shaanxi Province, China, **4** Department of Epidemiology and Biostatistics, Xi’an Jiaotong University School of Public Health, Xi’an, Shaanxi Province, China.
